# State of the art: 3D printing for creating compliant patient-specific congenital heart defect models

**DOI:** 10.1186/1532-429X-16-S1-W19

**Published:** 2014-01-16

**Authors:** Janelle Schrot, Todd Pietila, Anurag Sahu

**Affiliations:** 1 Biomedical Engineering, Materialise, Plymouth, Michigan, USA; 2 Cardiology, Emory University Hospital, Atlanta, Georgia, USA

## Background

Congenital heart defects affect 8 out of every 1,000 newborns. Although many do not require immediate treatment, most cases will require a surgical or catheter based intervention during their lifetime. Complex cases can involve multiple anomalies. These unique and complex procedures require comprehensive pre-intervention or surgical planning. A state of the art method for creating patient-specific compliant models of heart anatomy from medical image data is investigated. A case study is explored to assess the feasibility of this modeling method.

## Methods

**Figure 1:**
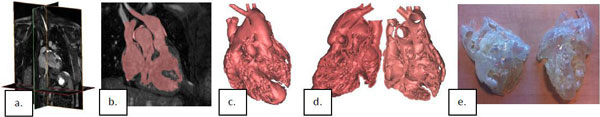
**Process for creating the model of the anomalous heart**. a. CMR images of the heart; b. Segmentation of the lumen; c. 3D reconstruction of the blood volume; d. Hollowed and sectioned view of the heart; e. 3D printed flexible heart.

This case study includes a patient with complx congenital heart disease consistenting of L-Transposition of the Great Arteries, subpulmonic stenosis, ventricular septal defect complicated by straddling tricusipid valve, and hypoplastic right ventricle. The uncorrected anatomy is being evaluated for surgical palliation. A cardiac magnetic resonance study and magnetic resonance angiography was obtained (Siemens Avanto 1.5T). Turbospin echo and gradient echo were used for anatomic definition. Following administration of gadolinium, volumetric acquisition of the chest was performed. The Mimics Innovation Suite software is then used to obtain virtual 3D reconstructed models of the heart, showcasing the defects, and to prepare them for 3D printing. Figure 1 shows the process for creating the physical heart model. The HeartPrint flex material process is used for 3D printing a multi-material compliant model of the patient-specific heart.

## Results

Flexible 3D printing with the HeartPrint flex material process was successful for representing patient-specific anomalies. Transparency and the ability to cut/bend the model were important features to include in the model for clinical inspection and assessment. All were achieved. The model was sectioned prior to printing to provide the most optimal viewing plane of the anomalies. Complex congenital heart disease presents unique challenges to cardiac imagers, interventional cardiologists, and cardiac surgeons. The relationship of the ventricular septal defect, size of the systemic ventricle, and presence straddling tricuspid valve tissue were important to understand. In this case the model provided accurate anatomical definition of complex cardiovascular structures. This allowed for a more complete assessment for various surgical options. The presence of hypoplastic right ventricle when viewed by the 3D model confirmed the findings of our 2D studies and moved us away from a two ventricle repair and towards a Glenn shunt and Fontan palliation.

## Conclusions

The HeartPrint process proved capable for timely providing clinicians with an accurate patient-specific model to aid in the pre-surgical planning for treatment of complex congenital heart defects. The use of 3D printing patient-specific models can be a powerful supplement to traditional imaging-only planning for complex congenital heart cases.

